# Event-Related Potentials Index Prediction Error Signalling During Perceptual Processing of Emotional Facial Expressions

**DOI:** 10.1007/s10548-023-00951-2

**Published:** 2023-03-14

**Authors:** Kristen S. Baker, Patrick Johnston, Naohide Yamamoto, Alan J. Pegna

**Affiliations:** 1 Faculty of Health, School of Psychology and Counselling, Queensland University of Technology (QUT), Brisbane, QLD Australia; 2 Defence Science and Technology Group, Information Sciences Division, Eagle Farm, QLD Australia; 3 Centre for Vision and Eye Research, Queensland University of Technology (QUT), Brisbane, QLD Australia; 4 Laboratory of Cognitive and Experimental Neuropsychology, Faculty of Health and Behavioural Sciences, School of Psychology, The University of Queensland, Brisbane, QLD Australia

**Keywords:** Attention, Emotion, Prediction, N170, N300

## Abstract

Humans use socially relevant stimuli to guide perceptual processing of the surrounding environment, with emotional stimuli receiving preferential attention due to their social importance. Predictive coding theory asserts this cognitive process occurs efficiently by combining predictions about what is to be perceived with incoming sensory information, generating prediction errors that are then used to update future predictions. Recent evidence has identified differing neural activity that demonstrates how spatial and feature-based attention may interact with prediction, yet how emotion-guided attention may influence this relationship remains unknown. In the present study, participants viewed a display of two faces in which attention, prediction, and emotion were manipulated, and responded to a face expressing a specific emotion (anger or happiness). The N170 was found to be enhanced by unpredictable as opposed to predictable stimuli, indicating that it indexes general prediction error signalling processes. The N300 amplitudes were also enhanced by unpredictable stimuli, but they were also affected by the attentional status of angry but not happy faces, suggesting that there are differences in prediction error processes indexed by the N170 and N300. Overall, the findings suggest that the N170 and N300 both index violations of expectation for spatial manipulations of stimuli in accordance with prediction error responding processes.

**Supplementary Information:**

The online version contains supplementary material available at 10.1007/s10548-023-00951-2.

## Introduction

Humans use their visual system to carry out a number of simultaneous tasks in order to function efficiently within the environment. One important area of interest in contemporary science is how the visual system accurately combines prior predictions with incoming sensory information. Over the last two decades, extensive literature has amassed in support of a unified framework of the mind, the theory of predictive coding. This theory argues the brain operates in a recursive hierarchical process, in which incoming stimulus input combines with the brain’s predictions, resulting in a residual of a prediction error signal. This prediction error signal is transmitted up levels in the cortical hierarchy for updating predictions to be used in future (Friston 2005; Rao and Ballard 1999). Attention is thought to contribute to this process by optimising the precision, or level of uncertainty, of this information (Clark 2013; Feldman and Friston 2010; Hohwy 2014). Attending to a stimulus usually enhances brain responses, which appears to conflict with the predictive coding theory as stimuli to be attended can be what the brain is prepared for, that is, more predictable. However, under a predictive coding framework attention may in fact operate by amplifying prediction error signals (Schröger 2016). Precision thereby is the measure of the prediction error signal’s reliability. In other words, attention operates by deciding whether the prediction error is signal rather than noise (Ransom et al. 2020). Furthering knowledge of how this ubiquitous process of attention fits into a predictive coding framework is important for understanding how the visual system processes the surrounding environment.

The predictive coding framework provides a plausible explanation for many of the interesting mechanisms that underlie cognitive processing in the brain. Yet, the specific mechanics of how this theory can explain the operation of cognitive processing of emotionally salient and attention-capturing information remains unclear. Indeed, information that is salient but not highly precise (i.e., it occurs with low probability and low reliability) can still capture attention (Ransom et al. 2020). Thus, it is challenging to explain enhanced responses to emotional stimuli under a precision optimisation account, if the brain’s main aim in predictive coding is to essentially minimise surprise (Ransom et al. 2020). Whilst it has been argued that the predictive coding theory is a unified framework (Clark 2013; Friston 2010), there is a need for more research to support this view, in particular into differences in perception guided by salient information, which occurs during emotion processing (Ransom et al. 2020). This is important to investigate as one of the key motivations linked to the evolution of human visual specialisation is attributed to sociality (Barton 2006), in which the social importance of emotions, communicated through facial expressions, plays a key role in communication.

The association between prediction and various types of attention, such as spatial and feature-based attention, generally indicates opposing and interactive relationships on a neurological level as supported by functional magnetic resonance imaging (fMRI) and electroencephalography (EEG) studies (e.g., Baker et al. 2022; Garrido et al. 2018; Jiang et al. 2013; Kok et al. 2012; Marzecová et al. 2017; Smout et al. 2019). An opposing relationship between attention and prediction is based on the observation that attention enhances prediction error signals to facilitate updating of priors. This is characterised as an opposing relationship because while attention increases neural activity, (correct) prediction exhibits the opposite effect by decreasing it. Recent studies have demonstrated that this occurs for both visual and auditory mismatch information elicited by stimuli which deviate from expectations (Auksztulewicz and Friston 2015; Garrido et al. 2018; Smout et al. 2019). On the other hand, the interactive view proposes that attention and prediction interact to produce greater neural activity when information is predictable and attended, so as to enhance the precision of predictions (Garrido et al. 2018; Hsu et al. 2014; Kok et al. 2012). This has been demonstrated in an fMRI study of spatial cueing in which predicted information enhanced brain activity in the visual cortex when attended but suppressed it when unattended (Kok et al. 2012). Another view indicates attention and prediction are distinct mechanisms that interact in a different manner during different stages of perceptual inference. This has been demonstrated in studies showing enhanced neural activity that differs between predicted and unpredicted stimuli at different latencies and scalp regions (Hsu and Hämäläinen 2021; Marzecová et al. 2017). Given the predictive coding framework argues prediction error units can arise from different cortical areas depending upon the context of the information (den Ouden et al. 2012; Friston 2005; Robinson et al. 2020b), it is important to examine which types of attention can interact with prediction to enhance neural activity as posited in previous studies of opposing and/or interactive effects. Thus, delving deeper into the relationship between attention and prediction specific to emotion-guided processing poses an interesting challenge.

### The N170

As it is evident that attention and prediction are important for producing a successful percept of the surrounding environment, the present study focused on two event-related potentials (ERPs) that have been found to be modulated by these processes – the N170 and N300. The N170 and N300 have been reported to be modulated by prediction error processes during visual perception, in particular by manipulating different aspects of stimuli, such as through spatial and feature-based attention (Allen-Davidian et al. 2021; Baker et al. 2021, 2022; Johnston et al. 2017; Kumar et al. 2021; Marzecová et al. 2017; Robinson et al. 2020a; Roehe et al. 2021; Tipples et al. 2013). Recent literature of the N170 has found it is sensitive to varied violations of visual prediction using faces, human statuettes, and geometric shapes (Allen-Davidian et al. 2021; Baker et al. 2021; Robinson et al. 2020a; Roehe et al. 2021). In our recent study investigating spatial attention in predictive coding, we found that the N170 and attention-sensitive N2pc were both enhanced when stimuli appeared in unpredictable spatial locations after following an expected trajectory, as compared to when they appeared in expected locations (Baker et al. 2021). The N1 has also been found to be enhanced by stimuli that were incongruent with an arrow’s direction, thus inducing expectation violations of location (Marzecová et al. 2017). The N170 is also sensitive to other non-spatially based violations. In one study that investigated ERP modulations as prediction error responses, a contextual trajectory paradigm was introduced (Johnston et al. 2017). This paradigm involves a sequence of events that are presented in a predicted manner until the endpoint, where the stimuli appear as either expected or unexpected. Johnston and colleagues (2017) used various manipulations to implement the paradigm, including rotating heads and bodies, shapes and faces moving around an imaginary central compass point, and faces morphing between neutral and happy expressions. Enhanced N170 amplitudes were found in response to unpredictable final images in comparison to their predictable counterparts, irrespective of the type of stimulus manipulation. This indicates the N170 is sensitive not just to spatial violations, but also to violations of other stimulus characteristics. Thus, it appears the N170 can be particularly sensitive to violations of predictions about changing spatial and feature-based attributes, but it remains ambiguous how emotion-guided attention affects prediction-related modulations of the N170 amplitude.

### The N300

The effects of prediction error responses to the N300 can be similar to those of the N170. This later occurring posterior-occipital N300 component has also been found to be enhanced when expectations are violated, such as relevant stimuli changing position (Johnston et al. 2017; Senju et al. 2006; Tipples et al. 2013), or incongruency in scene context (Kumar et al. 2021). However, unlike the N170, the N300 appears to be more sensitive to spatial, as opposed to feature-based, violations specifically (Baker et al. 2022; Johnston et al. 2017; Robinson et al. 2020b; Tipples et al. 2013). In our recent study, we presented shapes in a sequential trajectory in which they changed their position and/or shape at the final step. In this study, the N300 was found to increase to when the stimulus unexpectedly changed position, but this effect was not observed when the stimulus unexpectedly changed to a new shape (Baker et al. 2022). The N300 has also been found to be separable from the N170 in a similar trajectory violation paradigm, interestingly only for trials in which spatial attention needed to be reoriented to target location (Johnston et al. 2017). Other studies that manipulated spatial attention found larger N300 amplitudes when attention was redirected to the opposite side of the screen relative to where a target appeared, compared to when attention was focused on the same side as the targets (Senju et al. 2006; Tipples et al. 2013). Thus, the research evidence indicates that, like the N170, the N300 is sensitive to expectation violations. However, the N300 appears to be more so influenced by spatial violations, potentially reflecting a later-occurring updating of spatial predictions.

### Prediction Error and Emotion

Whilst there is empirical evidence that prediction error responses in visuospatial attention paradigms modulate evoked potentials, previous studies seldom investigated how emotion-directed attention affects modulations of the N170 and N300 by prediction errors. In one study investigating how emotion processing fits within a predictive coding framework, Vogel and colleagues (2015) argue that emotional stimuli amplify the prediction error response of another closely related visual evoked potential, the visual mismatch negativity (vMMN). This is evidenced by their study in which emotionally deviant faces demonstrated enhanced vMMN amplitudes compared to neutral counterparts (Vogel et al. 2015). This indicates that the relationship between attention and prediction needs to be considered in the context of not only physical and temporal properties, but also the potential emotional salience of the sensory information. This study provided initial promising insight into how emotion-based prediction errors may also modulate other early- to mid-latency visual evoked potentials. Indeed, sensitivity to emotional expression changes of the face is often considered to be first measured at the latency corresponding to the N170 within posterior regions of the brain. This N170 is considered to be the first component to index higher-level vision, because the preceding P1 is mainly sensitive to low-level visual feature changes (Johnston et al. 2017; Rossion and Caharel 2011). In support of the N170 sensitivity to higher-level vision, several studies have found that the N170 is sensitive to emotional changes in facial expressions (Blau et al. 2007; Brenner et al. 2014; Martin et al. 2021; Tian et al. 2018), with a meta-analysis identifying larger N170 amplitudes in response to negatively valanced faces compared to positively valanced happy faces; and furthermore, that it can be sensitive to both attended and unattended expressions (Hinojosa et al. 2015). More specifically, in relation to emotional salience, negatively valanced expressions (e.g., angry, sad, and fearful) tend to produce larger N170 amplitudes in comparison to positively valanced expressions, such as happy or joyful (Brenner et al. 2014; Liu et al. 2013; Tian et al. 2018). The overall findings of the N170 being modulated by both prediction and attention processes indicate that emotional salience may amplify the N170 prediction error response, in a way the same manner of amplification occurs in the closely related vMMN component (Vogel et al. 2015), with larger amplitudes elicited by angry than happy faces. By contrast, less is known about the N300’s sensitivity to emotional faces. If emotional stimuli do operate by enhancing prediction error responses, then they should amplify signals that signify spatial reorientation of attention, which the N300 appears to reflect. Thus, while it appears the N170 and N300 are sensitive to prediction error mechanisms, whether prediction-error modulations of the N170 and N300 interact or dissociate with emotion-guided prediction error remains an area for investigation.

### The Present Study

The sensitivity of the N170 and N300 visual evoked potentials to prediction error responses demonstrate they are valuable tools for studying electrophysiological processes of attention in the predictive coding framework. Thus, the present study investigated how the relationship between attention and prediction as reflected in prediction error responses may interact with emotional facial stimulus changes. This study examined whether prediction error signals such as the N170 and N300 can interact or dissociate with emotional prediction manipulations. As noted in several previous studies, these ERPs have been observed to be affected, in part, by manipulations of prediction and attention, in line with the proposal that they belong to a “family” of prediction error signals (Baker et al. 2021; Hohwy 2020; Johnston et al. 2017; Robinson et al. 2020a). Whilst these studies have identified interesting effects of spatial and feature-based attention on the ERPs as they were modulated by errors of prediction, how attention drawn to emotionally salient stimuli may interact with prediction remains ambiguous—specifically, whether emotion-guided attention further enhances or decreases neural activity that occurs through prediction error signals. To address this issue, the present study adapted the contextual trajectory paradigm and manipulated prediction and emotion through images of facial stimuli that gradually morphed from neutral to expressive angry and happy faces in multiple steps. This study also manipulated explicit attention by using salient red outlines around face stimuli preceding the final step in order to assess how emotion-guided attention differs from more exogenous and explicit guides of attention. If emotion-guided attention amplifies modulation of prediction error signals (Vogel et al. 2015), then it would be expected that enhanced N170 amplitudes would occur to unpredictable and attended manipulations (Johnston et al. 2017), and would also interact with expression, likely resulting in larger amplitudes in response to angry than happy expressions (Hinojosa et al. 2015). In addition, emerging evidence suggests the N300 may also reflect prediction error responses, likely as a result of spatial reorientation of attention that often co-occurs with prediction errors (Johnston et al. 2017; Senju et al. 2006; Tipples et al. 2013). If the N300 is a marker of spatial reorientation, then there would be different expectations for the effects of attention manipulations due to differential degrees of spatial reorientation required, such that larger amplitudes would occur to unpredictable and unattended stimuli as a larger spatial shift of attention is required, with the largest N300 in response to unpredictable and unattended stimuli, and the smallest N300 in response to predictable and attended stimuli. Furthermore, if emotional valance modulated N300 amplitudes, then an interactive effect of facial expression similar to that on the N170 would be observed, with larger amplitudes in response to angry than happy faces.

## Method

### Participants

Forty-three participants took part in the experiment. Four were excluded from analyses, due to artefacts in the EEG recording (*n* = 3) or having no correct behavioural responses (*n* = 1). Of the remaining 39 participants, their ages ranged from 17 to 60 years old (*M* = 21.26, *SD* = 7.30). Participants identified as female (*n* = 27) or male (*n* = 12) and were either right- (*n* = 35) or left-handed (*n* = 4). Participants were screened for normal or corrected-to-normal vision with no history of neurological disorders. Participants gave informed consent and received either university course credits or a gift card for their participation. The study was granted ethical approval by Queensland University of Technology’s Human Research Ethics Committee (approval number 1800000648).

### Design

**Fig. 1:**
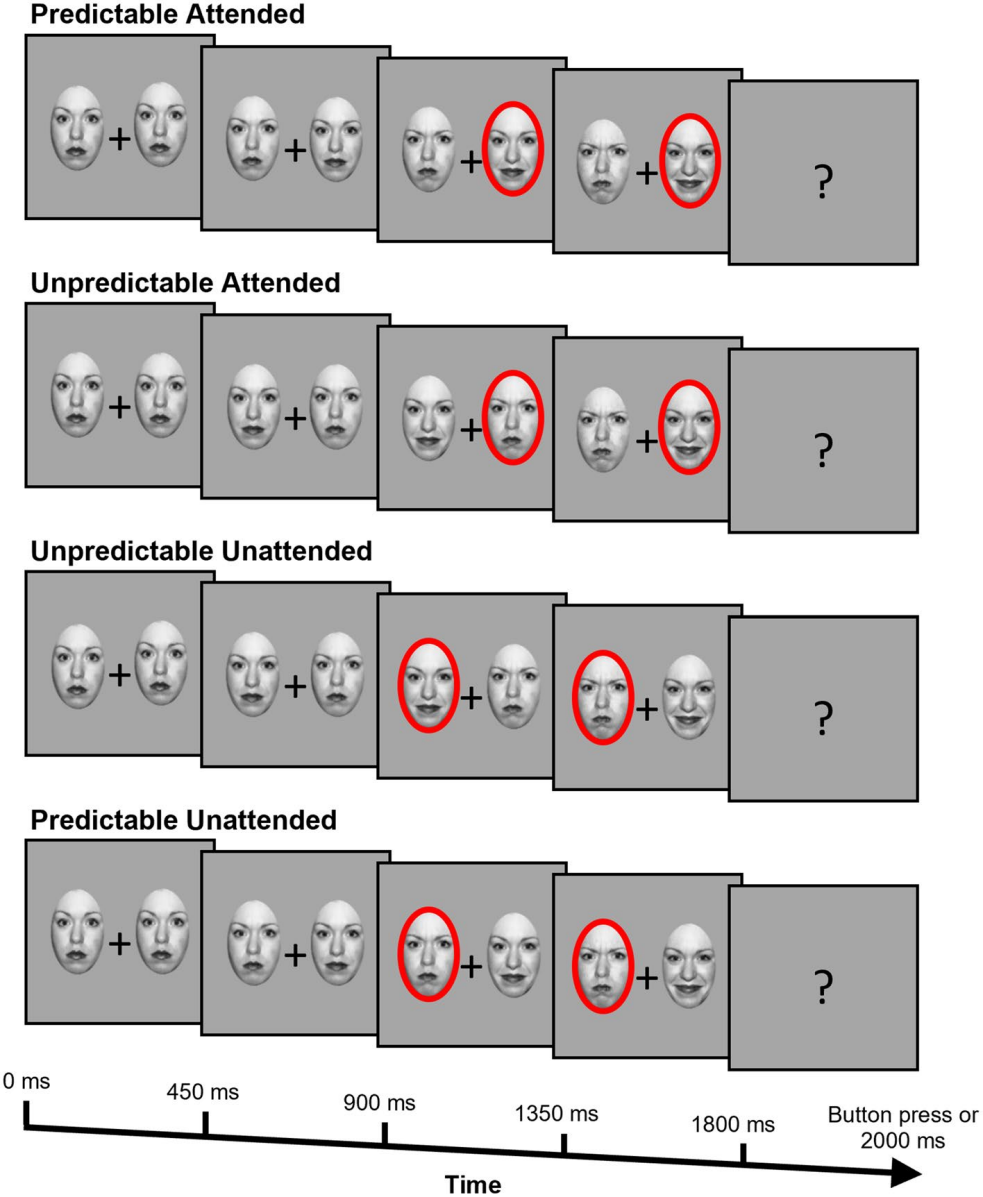
Example of one trial for four conditions pertaining to attention and prediction. In these examples, a female happy face is used as a target. In the experiment, the same four conditions were implemented using female angry, male happy, and male angry faces as well. Conditions named with respect to the status of the target in the final step. Each step containing face stimuli was displayed for 450 ms, and the prompt screen was displayed until participants’ response or 2000 ms elapsed. Stimuli not drawn to scale

Behavioural data consisted of percentage of correct responses and reaction times in a task in which participants detected a target face (details described below). For EEG responses, EEG recordings were time-locked to the onset of each stimulus presented. The relevant ERP signals were subsequently analysed by 2 × 2 × 2 × 2 repeated measures analyses of variance (ANOVAs), with factors of prediction (the same emotion appearing on the same versus opposite side in the final step as compared with preceding steps; Fig. 1), attention (attended versus unattended side containing red outline), target emotion (angry versus happy), and lateralisation (left versus right hemispheric activity) as described further below. The dependent variables were the mean amplitudes of the grand averaged ERPs of interest: N170 and N300.

### Instruments

#### Face Images 

Stimuli consisted of faces in neutral, angry, and happy expressions (Fig. 1). Angry and happy faces were chosen as exemplars of positively and negatively valanced stimuli. The stimuli were obtained from the NimStim Set of Facial Expressions (Tottenham et al. 2009). They were transformed into greyscale and presented on a medium grey background. The faces were morphed from neutral to angry, and from neutral to happy expressions in four steps. Facial morphs were created with Morpheus Photo Morpher software (Morpheus Morpheus Development 2014), by marking out key anatomical points of neutral and expressive face images, which the software then morphed together into separate images. As such, the angry stimuli consisted of 0% angry (i.e., 100% neutral), 33.3% angry, 66.6% angry, and 100% angry faces, with the same for happy stimuli. Two sequences of facial expressions were presented side-by-side (Fig. 1). Genders of the stimuli were counterbalanced throughout the gradations of emotions using one male and one female face. Images were cropped into an oval to include only facial features.

#### Immediate Mood Scaler

The Immediate Mood Scaler (IMS) is a 22-item questionnaire designed by Nahum and colleagues (2017). The purpose of the IMS in the present study was to measure each participant’s mood state at the time of the experiment. At the beginning of the experiment, participants rated on a 7-point Likert scale their current mood. Each item on the scale consisted of two descriptive mood words on either side of the scale, with ratings of 1 indicative of a lower mood (e.g., depressed or frustrated) and 7 indicating a higher mood (e.g., happy or peaceful). This questionnaire was used simply as a screening tool to exclude participants who were experiencing extreme mood states, which can influence ERP responses (Carboni et al. 2017; Cheng et al. 2017; Wang and Yang 2014; Wieser et al. 2010). However, as discussed below, no participants were excluded on the basis of their IMS scores.

### Procedure

Participants were seated approximately 60 cm from the computer and were provided with an information sheet and consent form to complete. Participants were presented with an information blurb describing the purpose of the IMS (see Supplementary Material Online Resource 1). Next, participants filled out the 22 items of the IMS via the online survey platform Qualtrics. Participants were fitted with a 64-channel EEG cap, corresponding to the 10–20 International system with a common reference (CMS/DRL). EEG recordings were measured at a sampling rate of 1024 Hz from 64 electrodes with an Active Two BioSemi Acquisition system (version 7.07, 2016). Participants viewed the stimuli on an HP liquid crystal monitor with a screen resolution of 1920 × 1080 pixels. Stimuli were displayed to participants on the monitor via the PsychoPy software (Version 2020.2.5; Peirce 2009). Participants were instructed to focus on a fixation cross at the centre of the screen as a series of images appeared on the screen and were informed to watch for a question mark to replace the cross. When the question mark appeared, they were to press the left or right arrow button on the keyboard to indicate where the target face appeared last. In the sequence each of the four face steps lasted for 450 ms, followed by a behavioural response screen that lasted until a response was made or 2000 ms was reached, before immediately preceding to the next sequence (Fig. 1). There were four blocks of trials, with a separate target image for each block. At the beginning of each block participants were informed of the target for that block. There were four possible targets: angry female, angry male, happy female, and happy male. All participants saw all stimuli, with the blocks presented in a random order for each participant. Within each of these blocks, all conditions were also presented in a random order, to minimise order effects.

The eight critical conditions were named with respect to prediction, attention, and target facial expression manipulations. Prediction was manipulated by having the first three steps of the sequence morph from a neutral to the expressive face. A predictable sequence was termed when the final (fourth) target stimuli followed the same morphing sequence on the side where the preceding three steps appeared. An unpredictable sequence was termed as such when the target and distractor stimuli swapped, so that at the fourth step the target appeared on the opposite side than as established in the three morphing steps prior. Attention was manipulated with a cue of a shaded red outline appearing on the third step and remaining on the same side for the fourth step in the sequence. This cue was to direct the attention of the participant to the face encircled by the shaded red outline, irrespective of whether this was the side in which the target emotion was being produced via morphing in the first three steps. The salient red cue was presented on the third rather than the fourth step, to ensure attention was not purely driven by the “pop out” effect occurring at the final step. An attended condition was termed as such when the red outline appeared on step 3 and the target appeared in the red outline on step 4. An unattended condition was termed as such when the target face was not red-outlined in step 4. Thus, in the predictable attended condition (abbreviated as PredAttend hereafter), the final target stimulus appeared on the same side as the same emotion morphing images in the first three steps, and in the same red outline as highlighted in step 3. In the predictable unattended condition (PredUnattend), the target stimulus appeared on the same side as in the predictable attended condition, but the red outline appeared on the opposing side around the distractor face. In the unpredictable attended condition (UnpredAttend), the target face in step 4 swapped to the side opposite to the preceding morphing steps, and appearing within the red outline. In the unpredictable unattended condition (UnpredUnattend), the target face swapped to the opposite side in the same manner but did not appear within the red outline. Within each of these four conditions, half the trials used angry targets and the other half used happy targets, creating the eight conditions. Furthermore, half of these angry targets appeared on the right side, and the other half on the left side, with the happy targets being shown in the same way. In total, participants viewed 960 trials with 70% of all trials being predictable and 30% of them being unpredictable. Half the predictable trials were attended, and the other half were unattended. The unpredictable trials were evenly divided in the same way.

### EEG Pre-processing and Analysis

Electrophysiological data were pre-processed using BrainVision Analyzer 2 (Version 2.1; Brain Products GmbH 2015), and ERP waveform processing and statistical analyses were done using MATLAB (Version 9.1; Mathworks 2019) and SPSS (Version 27; 2020), respectively. First, data were pre-processed by applying a bandpass filter of 0.1 to 30 Hz (24db/octave slope) with a notch filter of 50 Hz. Artefact rejection was performed by identifying segments that contained voltage fluctuations of 200 µV or greater and removing them with additional 100 ms periods immediately preceding and following these events. Overall, after performing artefact rejection there remained 34,763 trials (92.85% of the original trials), which entered subsequent analyses. More specifically, in each condition, the mean and standard deviation of trial numbers per participant were as follows: *M* = 310.74, *SD* = 39.18 (PredAttend); *M* = 312.56, *SD* = 37.35 (PredUnattend); *M* = 133.56, *SD* = 15.92 (UnpredAttend); and *M* = 134.49, *SD* = 15.41 (UnpredUnattend). When electrodes other than those used in the ERP analyses were noisy, they were interpolated using spherical spline interpolation. Eye-blinks were attenuated using the automated Independent Component Analysis procedure in BrainVision Analyzer 2 (Jung et al. 1998; Makeig et al. 1996). Data were re-referenced to the average of all electrodes. Separate epochs were generated for the final step of stimulus sequences using data from − 200 to 500 ms post final stimulus onset. Segments were separated by several factors including prediction (predictable and unpredictable), attention (attended and unattended), target emotion (angry and happy), lateralisation (left and right hemispheres), and face gender (male and female). Segments consisted of data for all trials such that trials of both correct and incorrect behavioural responses were included in the subsequent ERP analyses. Averages were generated for each epoch, and then baseline-corrected to the 150 ms period that immediately preceded the final stimulus onset. Grand averaged ERP waveforms were then produced for each electrode cluster, with the left cluster of electrodes P7, PO7, P5, and PO3, and the right cluster of electrodes P8, PO8, P6, and PO4. These electrodes were chosen a priori for ERP analysis as they were used in previous studies investigating similar ERP components in posterior occipital regions (Baker et al. 2021; Marzecová et al. 2017, 2018; Robinson et al. 2020a; Tipples et al. 2013). Pooled waveforms were generated by combining electrode activity across the left and right clusters.

To analyse each ERP component the mean amplitude was calculated within a relevant time window. For the N170 the peak-to-peak amplitude was extracted by subtracting the absolute maxima peak P1 values from the absolute minima peak N170 values for each condition and for each participant. The peak-to-peak analysis method ensured that in determining the size of the N170, differences in the preceding peak at the P1 latency were taken into account (Handy 2004). The P1 and N170 time windows were defined by taking time periods ± 10 ms relative to the peak latency (P1: 155 ms; N170: 200 ms). The N300 was measured between 250 and 350 ms, post final stimulus onset. Potential outlier participants were to be identified, if more than 10% of their grand averaged waveform activity during 450 ms post final stimulus onset was more than 2 *SD*s above or below the condition mean. However, no participants were excluded on this basis.

## Results

### Immediate Mood Scaler

The IMS responses for each participant were calculated by finding the mean score across all 22 items. On a scale ranging from 1 for low moods to 7 for high moods, participants’ overall mood ranged from 3.86 to 6.59 (*M* = 4.98, *SD* = 0.72). These results suggest that participants were experiencing a relatively average mood state on completion of the IMS at the beginning of the experiment. Thus, no participants were screened out on this basis. Detailed data are shown in Table S1 in Supplementary Material Online Resource 1.

### Behavioural Responses

Behavioural responses consisted of the percentage of accurate responses and reaction times to the target for all conditions (PredAttend, PredUnattend, UnpredAttend, and UnpredUnattend). Reaction times (in milliseconds) were recorded from the onset of the question mark prompting participants to respond as to which side the target face appeared last. Correct responses are named as such when participants answered the correct side in which the target face appeared last. Reaction times were calculated using correct responses only. Reaction time and accuracy statistics are displayed in Tables 1 and 2. These data are shown here for descriptive purposes only, as they were not relevant to the hypotheses of the present study (all of which were about modulation of ERP amplitude). That is, the behavioural task was used only for increasing participants’ engagement with the experiment by maintaining their gaze on a central fixation point. The assumption of sphericity was met for all ANOVAs (due to only two levels per each factor).

**Table 1:** Descriptive statistics: Reaction time of correct behavioural responses

Condition	Target emotion			
	Angry		Happy	
	*M*	*SD*	*M*	*SD*
PredAttend	385.31	100.08	369.98	100.66
PredUnattend	396.10	113.26	370.18	96.04
UnpredAttend	399.54	119.03	372.20	116.29
UnpredUnattend	390.98	118.83	381.07	131.47

Reaction time measured in milliseconds and calculated from correct responses only

**Table 2:** Descriptive statistics: Accuracy and error rates of all behavioural responses

Condition	Target emotion					
	Angry			Happy		
	Correct	Wrong	None	Correct	Wrong	None
PredAttend	95.71	2.21	2.08	96.38	1.74	1.88
PredUnattend	93.44	4.64	1.92	96.15	1.91	1.94
UnpredAttend	93.59	5.41	1.00	93.34	5.06	1.60
UnpredUnattend	91.13	6.98	1.89	93.09	5.59	1.32

Behavioural responses measured as percentages (%)

To determine whether there were significant differences in reaction times of correct responses, a 2 × 2 × 2 repeated measures ANOVA was performed with factors of prediction (predictable and unpredictable), attention (attended and unattended), and target emotion (angry and happy). This ANOVA revealed a significant three-way interaction, *F*(1, 38) = 5.78, *p* = .021, η_p_^2^ = 0.132. There was a statistically significant simple two-way interaction between prediction and attention in angry conditions, *F*(1, 38) = 5.51, *p* = .024, η_p_^2^ = 0.125, but not in happy conditions *F*(1, 38) = 1.11, *p* = .299, η_p_^2^ = 0.028. There was a statistically significant simple simple main effect of attention in angry conditions when stimuli were predictable *F*(1, 38) = 5,11, *p* = .030, η_p_^2^ = 0.119, but not when unpredictable, *F*(1, 38) = 1.28, *p* = .266, η_p_^2^ = 0.032. No other interactions or main effects reached significance in the omnibus ANOVA, all *F*s < 2.11, all *p*s > 0.155, all η_p_^2^s < 0.053. In sum, participants responded to predictable angry faces more quickly when they attended to them than when they did not, but otherwise the speed of response was largely equivalent among the conditions.

To analyse differences in accuracy, a 2 × 2 × 2 repeated measures ANOVA was performed with factors of prediction, attention, and target emotion. There were no significant interaction or main effects in the omnibus ANOVA, all *F*s < 2.53, all *p*s > 0.120, all η_p_^2^s < 0.06. This indicates there were no differences in the accuracy of responses between any conditions. Overall, participants performed the behavioural task with high accuracy in all conditions.

### ERP Analyses

#### N170

**Fig. 2:**
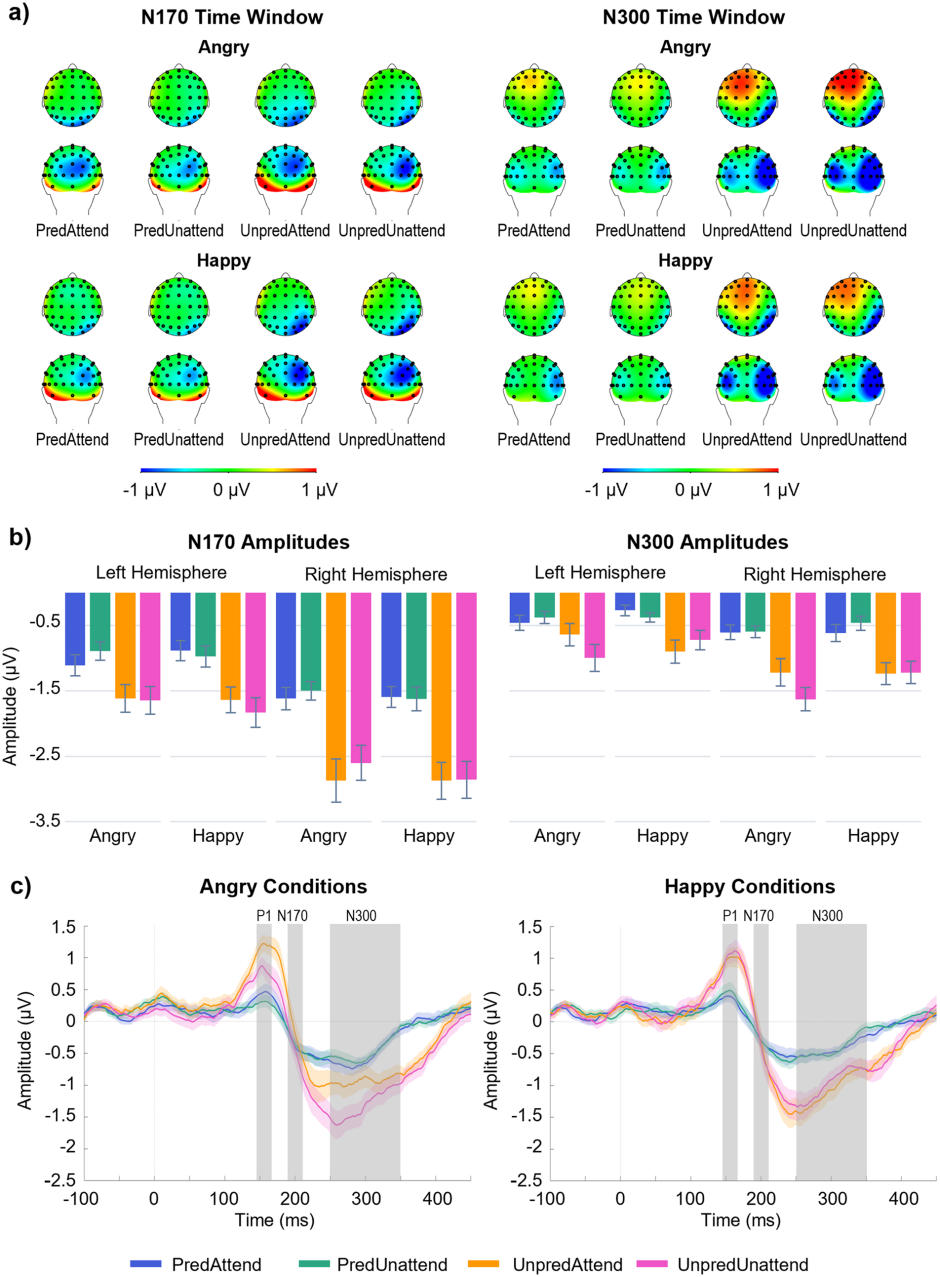
Mean N170 and N300 amplitudes as a function of prediction, attention, and emotion. (**A**) Scalp topographies of all conditions for N170 and N300 time windows. (**B**) Column graphs displaying N170 peak-to-peak and N300 mean amplitudes. Grey lines denote ± 1 standard error of the mean. (**C**) Grand averaged ERP waveforms pooled across both hemisphere electrode clusters, shown for each target emotion. Shading for each waveform represents ± 1 standard error of the mean at each time point

Visual depictions of N170 scalp topographies, mean amplitudes and grand averaged pooled waveforms are displayed in Fig. 2. A notable pattern observed in the data was that N170 amplitudes were larger (i.e., more negative) in unpredictable conditions, regardless of the target expressions. To statistically test these observations, a 2 × 2 × 2 × 2 ANOVA was performed with factors of prediction (predictable and unpredictable), attention (attended and unattended), target emotion (angry and happy), and lateralisation (left and right hemispheres). In addition, visual inspection of plots indicated a possible interaction between attention and emotion, but no interactions for prediction and lateralisation, which guided the tests that followed up the ANOVA.

The omnibus four-way ANOVA did not have a significant four-way interaction, *F(*1, 38) = 0.48, *p* = .491, η_p_^2^ = 0.013, or any three-way interactions, all *F*s < 2.04, *p*s > 0.162, η_p_^2^s < 0.051. There were significant two-way interactions between prediction and lateralisation, *F*(1, 38) = 7.26, *p* = .010, η_p_^2^ = 0.160, and between attention and emotion, *F*(1, 38) = 4.79, *p* = .035, η_p_^2^ = 0.112, as well as significant main effects of lateralisation, *F*(1, 38) = 18.46, *p* < .001, η_p_^2^ = 0.327, and prediction, *F*(1, 38) = 62.99, *p* < .001, η_p_^2^ = 0.624. No other main effects and interactions were significant, all *F*s < 2.04, all *p*s > 0.162, all η_p_^2^s < 0.051. The significant main effects showed that, overall, N170 amplitudes were larger when target faces were unpredictable (*M* = − 2.24 µV, *SE* = 0.19 µV) than predictable (*M* = − 1.27 µV, *SE* = 0.11 µV), and in the right hemisphere (*M* = − 2.19 µV, *SE* = 0.20 µV) than in the left hemisphere (*M* = − 1.32 µV, *SE* = 0.15 µV).

The interaction between attention and emotion occurred because there were larger N170 amplitudes in happy conditions when target faces were unattended than when they were attended, but the reverse pattern was found in angry conditions with larger amplitudes in attended than unattended conditions. The largest N170 amplitude was observed in happy unattended conditions (*M* = − 1.82 µV, *SE* = 0.16 µV), followed by angry attended conditions (*M* = − 1.80 µV, *SE* = 0.16 µV), happy attended conditions (*M* = − 1.74 µV, *SE* = 0.13 µV), and the smallest N170 in unattended angry conditions (*M* = − 1.66 µV, *SE* = 0.14 µV).

The interaction between prediction and lateralisation suggests that the effect of prediction might have been more pronounced in the right hemisphere than in the left hemisphere—that is, the difference between predictable and unpredictable conditions was larger in the right (*M*_diff_ = 1.22 µV) than left (*M*_diff_ = 0.72 µV) hemisphere (Fig. 2b). However, the effect of prediction was in the same direction in both hemispheres, and it remained significant when examined separately within each hemisphere: In the left hemisphere, unpredictable conditions (*M* = − 1.68 µV, *SE* = 0.18 µV) were significantly larger than predictable conditions (*M* = − 0.96 µV, *SE* = 0.13 µV), *t*(38) = 5.49, *p* < .001, Cohen’s *d* = 0.72; in the right hemisphere, unpredictable conditions (*M* = − 2.80 µV, *SE* = 0.27 µV) were also significantly larger than predictable conditions (*M* = − 1.58 µV, *SE* = 0.15 µV), *t*(38) = 7.03, *p* < .001, Cohen’s *d* = 0.90.

In sum, these results reveal that N170 amplitudes were generally larger in unpredictable conditions compared to predictable conditions, and in the right hemisphere than the left hemisphere. Although these two factors yielded a significant interaction, its pattern did not alter the above interpretations. Attention and emotion also subtly interacted, but the effect size of this interaction was much smaller than the effect sizes of the main effects of prediction and lateralisation. Taken together, the robust and theoretically relevant finding from the N170 analysis is that this ERP component was enhanced when targets were unpredicted.

#### N300

Scalp topographies, mean amplitudes, and grand averaged pooled waveforms for the N300 are depicted in Fig. 2. They showed that overall, amplitudes in unpredictable conditions were larger (i.e., more negative) than those in predictable conditions for both angry and happy faces. However, the two emotional faces also differed by the factors of prediction and attention, such that unattended angry faces yielded larger amplitudes than attended angry faces when they were unpredictable (but there was no such attentional effect when they were predictable), whereas happy faces did not cause any attentional modulation of the amplitudes regardless of their predictability. These observations were tested by the same 2 × 2 × 2 × 2 ANOVA as in the N170 analysis.

The omnibus four-way ANOVA showed there was not a significant four-way interaction, *F*(1, 38) = 1.01, *p* = .320, η_p_^2^ = 0.026, but it revealed there was a significant three-way interaction between prediction, attention, and target emotion, *F*(1, 38) = 6.83, *p* = .013, η_p_^2^ = 0.152, a significant two-way interaction between prediction and lateralisation, *F*(1, 38) = 4.53, *p* = .040, η_p_^2^ = 0.106, a significant two-way interaction between prediction and attention *F*(1, 38) = 4.65, *p* = .037, η_p_^2^ = 0.109, a significant two-way interaction between attention and emotion, *F*(1, 38) = 4.94, *p* = .032, η_p_^2^ = 0.115, a significant main effect of lateralisation *F*(1, 38) = 11.16, *p* = .002, η_p_^2^ = 0.227, and a significant main effect of prediction, *F*(1, 38) = 40.97, *p* < .001, η_p_^2^ = 0.519. The other interactions and main effects were not significant in the omnibus ANOVA, all *F*s < 4.00 all *p*s > 0.053, all η_p_^2^s < 0.095.

To follow up the significant three-way interaction, a simple two-way interaction between prediction and attention was tested separately in angry and happy conditions. Prediction and attention significantly interacted in the angry conditions, *F*(1, 38) = 4.95, *p* = 0.032, η_p_^2^ = 0.115, but not in the happy conditions, *F*(1, 38) = 0.15, *p* = 0.700, η_p_^2^ = 0.004. The significant simple two-way interaction between prediction and attention was further followed up by testing a simple simple effect of attention separately in predictable and unpredictable conditions. This effect was significant in the angry unpredictable conditions, *F*(1, 38) = 7.81, *p* = 0.008, η_p_^2^ = 0.171, but not in the angry predictable conditions, *F*(1, 38) = 0.12, *p* = 0.728, η_p_^2^ = 0.003. These follow-up test results were in line with the observations noted above, and they also help explain the significant omnibus two-way interactions involving prediction, attention, and emotion. That is, these omnibus interactions became significant because the effects of attention appeared differently depending on whether target faces were predictable or unpredictable, and also whether they expressed angry or happy emotion.

Regarding the omnibus effects involving the lateralisation factor, generally, the N300 amplitude was larger in the right hemisphere (*M* = − 0.95 µV, *SE* = 0.11 µV) than in the left hemisphere (*M* = − 0.59 µV, *SE* = 0.10 µV). The prediction × lateralisation interaction occurred because the effect of prediction was in the same direction in both hemispheres (i.e., unpredictable target faces evoking larger N300s) but greater in the right (*M*_diff_ = 0.75 µV) than left (*M*_diff_ = 0.44 µV) hemisphere. Thus, the hemispheric difference was notable, but it did not alter the conclusions about the effects of attention, prediction, and emotion.

In sum, these results provide partial support for the hypothesis that the N300 would be largest in unpredictable and unattended conditions. That is, this pattern was only identified for angry conditions—the largest N300 amplitudes occurred in UnpredUnattend conditions (*M* = − 1.31 µV, *SE* = 0.16 µV), followed by UnpredAttend conditions (*M* = − 0.93 µV, *SE* = 0.14 µV), PredAttend conditions (*M* = − 0.53 µV, *SE* = 0.10 µV), and the smallest N300 in PredUnattend conditions (*M* = − 0.48 µV, *SE* = 0.08 µV). In happy conditions, regardless of attention, unpredictable conditions elicited larger N300 amplitudes (*M* = − 1.02 µV, *SE* = 0.13 µV) than predictable conditions (*M* = − 0.43 µV, *SE* = 0.07 µV).

## Discussion

Under the perspective of a predictive coding framework, the aim of the present study was to investigate how prediction interacts with attentional salience of stimuli using manipulations of separable attention and emotion-guided cues. To achieve this, the present study used a contextual trajectory paradigm and investigated ERP components that have been found to index prediction and attention processes: the N170 and N300. Overall, it was expected that N170 amplitudes would be sensitive to prediction violations; and furthermore, that the N300 would follow a spatial reorientation hypothesis, in which larger N300 amplitudes would occur when a larger shift of spatial attention was required. It was also predicted that if emotion-guided attention amplifies prediction error signals then an interaction with emotion would be observed for the N170 and N300 responses, likely with larger amplitudes to angry than happy expressions.

For N170 amplitudes, a two-way interaction was evident between attention and emotion, but the small effect size suggests this was likely due to subtle (and therefore negligible) variations of N170 amplitudes according to attention and target emotion that occurred on top of the effects of prediction and lateralisation. Hence, the main findings were that the N170 was consistently larger for unpredictable than predictable stimuli, and in the right hemisphere than left hemisphere. Previous research investigating ERP components for studying prediction error responses has found robust evidence that the N170 is enlarged by expectation violations (Allen-Davidian et al. 2021; Baker et al. 2021, 2022; Johnston et al. 2017; Marzecová et al. 2017; Robinson et al. 2020a; Roehe et al. 2021). These previous studies manipulated different characteristics of stimuli of geometric shapes, faces, and bodies, and thus there is an unambiguous demonstration of the N170 being modulated by prediction error signalling, regardless of attentional salience that is intrinsic to the stimuli. Indeed, in the present study, the N170 did not appear to be largely affected by preference for negatively or positively valanced target stimuli. Instead, the attention and emotion effects were separable from those of prediction errors. Interestingly, Johnston and colleagues (2017) also investigated prediction errors in processing morphing of facial expressions, and they did not find any differences between neutral and expressive faces in the N170 and N300. It appears, then, that akin to the present study, the N170 is sensitive to violations of expectations rather than attentional and emotional aspects of the target stimulus. Thus, these findings as well as the effect of predictability indicate that in the presence of prediction error signals, the N170 broadly responds to early instances of these signals, which are modulated by expectation violations, irrespective of the type of attentional conditions of stimuli such as red coloured cues or target emotion.

Turning to the findings of lateralisation effects on the N170, they have also been identified in a similar study of a contextual trajectory paradigm that used variations of facial stimuli in lightning, orientation, and the direction of gravitational pull (Allen-Davidian et al. 2021). Similar to the present findings, there appeared to be greater activity in the right hemisphere compared to the left. Likewise, Johnston and colleagues (2017) found effects of lateralisation for the N170 that did not interact with predictability of stimuli, with larger activity in the right hemisphere than the left. Together with the current finding that the effect of prediction occurred in the same direction in both hemispheres, these results suggest that the N170 can be larger in the right hemisphere, but this does not qualitatively alter the effects of expectancy violations. Overall, then, it appears the N170 is largely reflective of prediction error signalling, but this is dissociated from lateralisation effects.

On the basis of the predictive coding responses observed in previous studies, we formulated the spatial reorientation hypothesis of the N300, which predicted larger N300 amplitudes when a larger spatial reorientation of attention was required, as was the case in the present study for unpredictable conditions compared to predictable conditions. Furthermore, in angry conditions the prediction factor interacted with attention, such that in line with our hypothesis, UnpredUnattend conditions elicited the largest mean N300 amplitude, which was reliably greater than the second largest mean N300 amplitude yielded by UnpredAttend conditions; and in contrast to the hypothesis, PredAttend and PredUnattend conditions produced statistically equivalent mean N300 amplitudes, the former of which was hypothesised to evoke the smallest N300. Hence, the hypothesis was partially supported in that the condition that required the largest redirection of spatial orientation showed the greatest N300 amplitude; however, this pattern was observed only in angry conditions, and even when target faces were angry, the condition that involved the smallest degree of spatial reorientation did not show the smallest N300 amplitude.

The N300 as a marker of spatial reorientation of attention has been demonstrated in previous studies in which invalid cues directing attention to a task-irrelevant location elicited larger N300 amplitudes than valid cues (Johnston et al. 2017; Senju et al. 2006; Tipples et al. 2013). In further support of this, in a magnetoencephalographic study, a mid-latency prediction error response that is thought to correspond to the N300 component has been found to occur in the right supramarginal gyrus (Robinson et al. 2020b) a region that is often considered responsible for spatial attention processing (Loayza et al. 2011; Silk et al. 2010). These previous findings support the current findings that the N300 was sensitive to the spatial manipulations of target predictability, with larger amplitudes when a larger spatial reorientation to the opposite side of the screen was required as occurred in unpredictable conditions. The present study provides a new insight into the spatial reorientation process as indexed by the N300, indicating that the valence of the stimulus can modulate the amplitude, as attending to angry stimuli reduced the neural activity of unpredictability-driven spatial reorientation, whereas attending to happy stimuli did not induce this change. This suggests that the N300 amplitude does index spatial reorientation, but the exact patterns of its modulation can vary according to the emotional valence of stimuli because differentially valanced stimuli can lead to differential degrees of attentional reorientation (Feldmann-Wustefeld et al. 2011; Holmes et al. 2009).

As described previously, the function of attention in predictive coding is thought to be the optimisation of precision, or reliability, of signals (Clark 2013; Feldman and Friston 2010; Hohwy 2014). As predictable conditions occurred with 70% probability, and the remaining 30% probability consisting of unpredictable conditions, higher precision was expected in predictable conditions. Deviation away from a high precision state (i.e., unpredictable conditions) is thought to lead to a larger prediction error signal (Clark 2013; Hohwy 2014; Robinson et al. 2020a). It has been argued that the salience of information, such as unpredictability of stimuli, interacts with bottom-up perceptual information so that it becomes critical to pay attention to salient prediction error to minimise long-term uncertainty (Clark 2018; Parr and Friston 2017). The findings of the present study have demonstrated that salience of attention cues and emotional expressions does not appear to interact with the N170 prediction error responses. On the other hand, the N300 results indicate prediction error responses can interact with attention cues during spatial reorientation, for angry but not happy faces. As per previous studies, different ERP components have been found to index distinct stages of the interaction between prediction and attention, such that the latency and region of electrodes each reflects different neural activities that would underlie different cognitive processes (Hsu and Hämäläinen 2021; Marzecová et al. 2018). As such, these early- to mid-latency evoked potentials may represent the initial flood of prediction error that registers deviation from an expected state (N170), which is then later examined in detail by spatially reorienting attention as necessary (N300) (Clark 2013; Johnston et al. 2017).

To date, research investigating modulations of visually evoked potentials by prediction error has focused predominantly on spatial, feature-based, or object-based manipulations. These studies have provided important empirical evidence that early- to mid-latency potentials can be modulated by prediction error responses. Yet, it remained ambiguous how emotion-guided attention can influence prediction error responses, under a predictive coding framework. This study aimed to elucidate whether emotion-guided attention could enhance prediction error responses. The present findings did not show emotion effects that interacted with predictability. Rather, they support the view that the N170 can be considered as a generic prediction error signal, which is largely modulated by expectation violations in general, being invariant to attentional or emotional salience of stimuli. The present findings also support an emerging view that the N300 may reflect spatial reorientation of attention following a prior spatial expectation violation, and provide a new insight into how the (angry) emotion of the target can modulate this response.

However, several limitations of the present study should be noted. Firstly, while the study aimed to elucidate the influence of emotion and attention on prediction error signalling, the prediction was largely defined spatially and thus it can only offer a perspective of the manipulations of spatial expectations. Interestingly, a possible additional effect of spatial reorientation was observed as a frontal positivity in the N300 time window (see the topographies in Fig. 2a), suggesting differences between predictable and unpredictable conditions. Given the focus of the present study was on posterior N300 effects, no attempt was made to interpret the frontal effect. It should be further investigated in future research. Secondly, while it was important to morph the emotional stimuli to form a predictable contextual trajectory, the influence of the emotional manipulation may have become subtle by the time the fourth step was reached. This was because the emotional expressions changed in increments of 25%, such that at the third step the emotion had already been displayed with 75% intensity. Although these effects would have been applicable to both angry and happy trajectories, the lack of an attention effect on happy faces may have been because they were not particularly salient by the time the facial expression reached the 100% intensity, in comparison to angry faces at 100% intensity. Furthermore, the morphing manipulation may have also influenced the ERP amplitudes, as variations in ERP responses to transient events can cause multiple components to overlap in time windows (as discussed in Rossion 2014), and make amplitudes smaller as the steps of contextual trajectories transpire (Johnston et al. 2017). This could possibly explain why the effects of the N170 in the typical time window were present on the downward slope of the N300, rather than at the peak of the first negative component, as observed in Fig. 2c. Future research would benefit from maintaining the salience of the expression throughout the expression intensity steps, perhaps by using images of different identities, a faster trial period, or presenting control images between each image presentation.

## Conclusion

The present study investigated neural activity that is thought to index the relationship between prediction and attention, through the manipulation of attentional cues and emotions of stimuli. Overall, the findings of this study provide support for the N170 and N300 as early- to mid-latency prediction error signals, modulated by expectation violations but largely uninfluenced by attention or emotion changes. The N170 was found to be enhanced by unpredictable as opposed to predictable stimuli, indicating that it indexes general prediction error signalling processes. The N300 amplitudes were also enhanced by unpredictable stimuli, but they were also affected by the attentional status of angry but not happy faces, suggesting that there are differences in prediction error processes indexed by the N170 and N300.

## Supplementary Information

Below is the link to the electronic supplementary material.

Supplementary Material 1 (DOCX 22.0 kb)

## Data Availability

The datasets generated and analysed during the current study are available in the Open Science Framework repository, https://osf.io/qxvt5/.
