# Supplementary material for: Event-Related Potentials Index Prediction Error Signalling During Perceptual Processing of Emotional Facial Expressions
Source: Brain Topogr. 2023 Mar 14;36(3):419–32. doi: 10.1007/s10548-023-00951-2 (PMC10164013; doi:10.1007/s10548-023-00951-2)
Supplement: Supplementary file 1 — Supplementary Material 1 (DOCX 22.0 kb) [file 10548_2023_951_MOESM1_ESM.docx]

Supplementary Material

**Article Title**

Event-Related Potentials Index Prediction Error Signalling during Perceptual Processing of Emotional Facial Expressions

**Journal Name**

Brain Topography.

**Author Names and Affiliations**

Kristen S. Baker^1^, Patrick Johnston^2^, Naohide Yamamoto^1,3^, and Alan J. Pegna^4^

^1^ School of Psychology and Counselling, Faculty of Health, Queensland University of Technology (QUT), Brisbane, Queensland, Australia

^2^ Defence Science and Technology Group, Information Sciences Division, Eagle Farm, Queensland, Australia

^3^ Centre for Vision and Eye Research, Queensland University of Technology (QUT), Brisbane, Queensland, Australia

^4^ Laboratory of Cognitive and Experimental Neuropsychology, School of Psychology, Faculty of Health and Behavioural Sciences, The University of Queensland, Brisbane, Queensland, Australia

**Correspondence**

Correspondence concerning this article should be addressed to Alan Pegna, Laboratory of Cognitive and Experimental Neuropsychology, School of Psychology, Faculty of Health and Behavioural Sciences, The University of Queensland, Brisbane, Queensland, Australia. Email: a.pegna@uq.edu.au

Online Resource 1

Information Blurb for the Immediate Mood Scaler

The following text was presented to participants prior to commencing the Immediate Mood Scaler:

“Thank you for contributing to this research. This part involves completing one questionnaire. The questionnaire is designed to measure immediate mood states, in particular whether you are more or less likely to be feeling a particular mood at the time of participation. The questionnaires are not clinical diagnostic tools, but just research instruments in this study, to get information about where people sit on the range of mood experience during study participation. Your responses do not indicate “normality” or “abnormality” in current mood states. In other words, indicating the presence or absence of particular types of moods in these questionnaires should not make you concerned about your mental health. Nevertheless, if you experience discomfort or distress as a result of answering the questionnaires, please note that you have access to a free counselling service at QUT Psychology and Counselling Clinic, as detailed in the Participant Information Sheet.”

**Table S1.**
Mean Immediate Mood Scaler responses for all included participants (N = 39)

| Question | Item | *M* | *SD* |
| --- | --- | --- | --- |
| 1 | Depressed (1) – Happy (7) | 5.18 | 1.19 |
| 2 | Distracted (1) – Focused (7) | 4.79 | 1.17 |
| 3 | Worthless (1) – Valuable (7) | 5.56 | 1.02 |
| 4 | Lonely (1) – Engaged (7) | 4.95 | 1.27 |
| 5 | Sleepy (1) – Alert (7) | 4.46 | 1.47 |
| 6 | Slow (1) – Speedy (7) | 4.42 | 1.18 |
| 7 | Tired (1) – Energetic (7) | 4.05 | 1.21 |
| 8 | Pessimistic (1) – Optimistic (7) | 5.23 | 0.90 |
| 9 | Apathetic (1) – Motivated (7) | 5.33 | 1.08 |
| 10 | Guilty (1) – Proud (7) | 4.79 | 1.03 |
| 11 | Numb (1) – Interested (7) | 5.90 | 0.97 |
| 12 | Withdrawn (1) – Welcoming (7) | 5.30 | 0.98 |
| 13 | Frustrated (1) – Peaceful (7) | 5.38 | 1.35 |
| 14 | Impulsive (1) – Careful (7) | 5.18 | 1.17 |
| 15 | Moody (1) – Stable (7) | 5.03 | 1.16 |
| 16 | Hopeless (1) – Hopeful (7) | 5.46 | 0.88 |
| 17 | Irritable (1) – Easy-going (7) | 5.46 | 1.39 |
| 18 | Tense (1) – Relaxed (7) | 4.44 | 1.50 |
| 19 | Worried (1) – Untroubled (7) | 4.49 | 1.23 |
| 20 | Fearful (1) – Fearless (7) | 4.92 | 0.97 |
| 21 | Anxious (1) – Peaceful (7) | 4.41 | 1.46 |
| 22 | Restless (1) – Calm (7) | 4.90 | 1.45 |
| Total |  | 4.98 | 0.72 |

*Note*. The Immediate Mood Scaler (Nahum et al., 2017) consists of 22 items, in each of which participants rate their current mood on a 7-point Likert scale that is defined by two opposing moods (e.g., depressed and happy).
